# Polyaniline-Coated Activated Carbon Aerogel/Sulfur Composite for High-performance Lithium-Sulfur Battery

**DOI:** 10.1186/s11671-017-2372-6

**Published:** 2017-12-12

**Authors:** Zhiwei Tang, Jinglin Jiang, Shaohong Liu, Luyi Chen, Ruliang Liu, Bingna Zheng, Ruowen Fu, Dingcai Wu

**Affiliations:** Materials Science Institute, PCFM Lab and GDHPRC Lab, School of Chemistry, Sun Yat-sen University, Guangzhou, 510275 People’s Republic of China

**Keywords:** Activated carbon aerogel, Hierarchical porous carbon, Polyaniline coating, Lithium-sulfur battery

## Abstract

An activated carbon aerogel (ACA-500) with high surface area (1765 m^2^ g^−1^), pore volume (2.04 cm^3^ g^−1^), and hierarchical porous nanonetwork structure is prepared through direct activation of organic aerogel (RC-500) with a low potassium hydroxide ratio (1:1). Based on this substrate, a polyaniline (PANi)-coated activated carbon aerogel/sulfur (ACA-500-S@PANi) composite is prepared via a simple two-step procedure, including melt-infiltration of sublimed sulfur into ACA-500, followed by an in situ polymerization of aniline on the surface of ACA-500-S composite. The obtained ACA-500-S@PANi composite delivers a high reversible capacity up to 1208 mAh g^−1^ at 0.2C and maintains 542 mAh g^−1^ even at a high rate (3C). Furthermore, this composite exhibits a discharge capacity of 926 mAh g^−1^ at the initial cycle and 615 mAh g^−1^ after 700 cycles at 1C rate, revealing an extremely low capacity decay rate (0.48‰ per cycle). The excellent electrochemical performance of ACA-500-S@PANi can be attributed to the synergistic effect of hierarchical porous nanonetwork structure and PANi coating. Activated carbon aerogels with high surface area and unique three-dimensional (3D) interconnected hierarchical porous structure offer an efficient conductive network for sulfur, and a highly conductive PANi-coating layer further enhances conductivity of the electrode and prevents the dissolution of polysulfide species.

**Electronic supplementary material:**

The online version of this article (10.1186/s11671-017-2372-6) contains supplementary material, which is available to authorized users.

## Background

The development of portable electronics, electric vehicles (EVs), and smart grid systems has been continuously demanding rechargeable batteries with high energy density, long life span, and low cost. Lithium-sulfur (Li-S) batteries have become one of the most promising candidates for next-generation lithium secondary batteries due to their high theoretical capacity (1675 mAh g^−1^) and theoretical energy density (2600 Wh kg^−1^, 2800 Wh l^−1^, respectively). Furthermore, from a practical perspective, element sulfur is naturally abundant, low cost, nontoxic, and environmentally friendly compared with other traditional transition metal oxide cathode materials [1–3]. In spite of these advantages, practical applications of Li-S batteries are hindered owing to the following critical issues which lead to low active material utilization and poor cyclability: (1) low electronic and ionic conductivity of sulfur (5 × 10^−30^ S cm^−1^) and its discharge products Li_2_S/Li_2_S_2_ [4]; (2) severe dissolution of intermediate polysulfides in electrolyte, forming the so-called shuttle effect [5]; and (3) large volumetric expansion (~ 76%) during the discharge of cell [6]. In recent years, enormous efforts have been made to improve the performance of sulfur-based cathodes, including using various inorganic/organic conducting substrates [7–9], modifying separator/lithium anode [10, 11], and optimizing binder [12]/electrolytes [13] for enhancing the conductivity of the cathode, mitigating the diffusion of polysulfides, and accommodating volume expansion.

Among various kinds of conducting substrates, porous carbon materials have attracted great attention due to their high surface area, good conductivity, and excellent electrochemical stability. Recent progresses in carbon/sulfur cathodes have revealed that porous carbon serving as host material for sulfur can effectively overcome the above drawbacks and demonstrated increased cycling stability [14–16]. Carbon aerogels (CAs) owned moderate surface area, and 3D interconnected hierarchical porous nanonetworks are considered to be ideal candidates for sulfur host material. Yin et al. reported that CAs with abundant narrow micropores could be utilized as an immobilizer host for sulfur impregnation and obtained preferable reversible capacity and excellent cycling stability for S/CA hybrid cathodes [17]. Fang et al. synthesized multifarious CAs by ambient pressure drying with different ratios of raw materials and discussed their Li-S battery performance [18]. However, all of the above used CAs’ own relatively low specific surface area (below 700 m^2^ g^−1^) and small pore volume (below 1.1 cm^3^ g^−1^), which limited sulfur loading and impaired the capacity of carbon/sulfur composite.

In order to increase sulfur loading to improve electrochemical performance, high pore volume and specific surface area of carbon aerogels are needed. Currently, chemical activation is an important method to increase pore volume and surface area of carbon materials. However, it is still a challenge for obtaining high surface area and large pore volume of activated carbon under a low activation ratio in order to decrease the consumption of activation agent and reduce environmental pollution. Moreover, carbon aerogels are usually consisted of relatively large mesopores, which are unfavorable for sequestering sulfur. Therefore, in a high sulfur loading condition, it is more important to solve the problem of sulfur dissolution. Recently, some studies had shown that the capacity and cycling stability of carbon/sulfur composite could be further improved by using a conductive coating, such as graphene [19], reduced graphene oxide [20], and conducting polymers including polyaniline (PANi) [21, 22], polypyrrole (PPy) [23], and poly(3,4-ethylenedioxythiophene)-poly(styrene sulfonate) (PEDOT:PSS) [24]. Conductive shell on the external surface of a carbon/sulfur composite not only held and trapped polysulfide species to minimize the active material loss, but also offered a shorter path length for ion and electron transport and further improved the conductivity of the composite, leading to better reaction kinetics and enhanced rate performance. As a common conducting polymer, polyaniline had been widely used to improve the electrochemical properties of supercapacitors [25], chemosensors [26], and lithium ion/fuel cells [27, 28] as either a conductive matrix or a soft modified framework on the carbon surface due to its facile synthesis process, scale-up, self-healing, relatively high electrical conductivity, intrinsic ample nitrogen functional groups, and environmental stability.

**Scheme 1:**
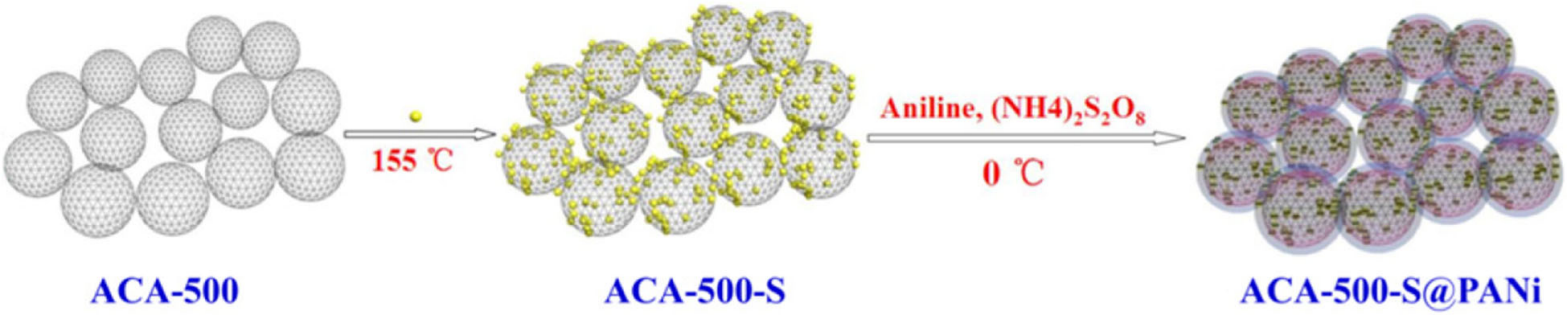
Schematic illustration for the preparation of ACA-500-S@PANi

In this work, we adopt activated carbon aerogels (ACA-500) as an immobilizer host for sulfur impregnation, which are prepared by KOH activation of organic aerogel (RC-500) at a low ratio of 1:1 (as shown in Scheme 1). ACA-500 owns a high surface area (1765 m^2^ g^−1^), large pore volume (2.04 cm^3^ g^−1^), and hierarchical porous nanonetwork, and the ACA-500-S composite contained relatively high sulfur content (63%). Furthermore, polyaniline is used to coat on the surface of the ACA-500-S composite by in situ chemical oxidation polymerization to prevent active sulfur and polysulfide species from dissolving in the electrolyte. The results indicate that ACA-500-S@PANi composites present much more superior initial capacity and cycle stability compared to the uncoated ACA-500-S and other coating composites presented in the literatures, due to the existence of the polyaniline-coating layer.

## Methods/Experimental

### Sample Preparation

RF organic aerogels and activated carbon aerogels were prepared according to the method described previously [29]. Detailed procedure were as follows: All reactants with precalculated formulations(500:1 molar ratio of R:C), including resorcinol (R), formaldehyde (F), deionized water (W), and cetyltrimethylammonium bromide (C), were transferred into a glass vial (20 ml) and mixed by a magnetic stirrer at room temperature. Then, the vial was sealed and put into a water bath (85 °C) to cure for 5 days. After curing, the gels were directly dried for 24 h at room temperature in air, 24 h at 50 °C, and 3 h at 100 °C at ambient pressure in sequence. Subsequently, the resultant RF organic aerogels were annealed at 500 °C for 3 h with a heating rate of 5 °C min^−1^ under N_2_ flow (400 ml min^−1^), obtaining RC-500-S500. Activated carbon aerogels (ACA-500) were prepared according to the following procedures [30]: Approximately 2 g of RC-500-S500 were mixed with potassium hydroxide (KOH) at a mass ratio 1:1 in a glass beaker, and 10~15 ml ethanol was added to dissolve the KOH. The mixture was dried at 110 °C, then carbonized in a tubular furnace at 900 °C for 3 h with 5 °C min^−1^ under N_2_ flowing (400 ml min^−1^). After cooling down to room temperature, the resultant materials were taken out and washed with 10% HCl solution and distilled water. Finally, the materials were dried at 110 °C for 6 h. The resulting products were referred as ACA-500.

ACA-500-S composites were prepared by heating-melting of a mixture of ACA-500 and elemental sulfur (mass ratio = 3:7). ACA-500-S@PANi composites were prepared by in situ chemical oxidative polymerization at the freezing temperature on the surface of the ACA-500-S composite, as described in the literature [31]. Typically, the ACA-500-S composite (0.2 g) was dispersed in the distilled water/acetone (27 ml/3 ml) mixed solution by ultrasound. Then, aniline monomer (0.28 g) and 1 M HCl solution (15 ml) were added into it, and the mixture was stirred vigorously for 15 min at 0 °C. Subsequently, a precooled aqueous solution of (NH_4_)_2_S_2_O_8_ (0.123 g dissolved in 30 ml distilled water) was added dropwise into the above reactant solution. After constant reaction for 6 h under stirring, the precipitate was filtered and washed with distilled water and ethanol until the filtrate became transparent. Then, the product was dried in a vacuum oven at 50 °C overnight to obtain ACA-500-S@PANi. The sulfur content in the as-prepared ACA-500-S and ACA-500-S@PANi composites was calculated by thermogravimetric analysis (TGA). Similarly, we prepared ACA-500-S composites with 70 and 54% sulfur contents under 1:3 and 2:3 mass ratio of ACA-500 and sulfur (denoted as ACA-500-S-70% and ACA-500-S-54%, respectively). ACA-500-S@PANi composites with 45, 55, and 61% sulfur contents were prepared from ACA-500-S-54% and ACA-500-S-70% under mass ratio of aniline monomers to C/S composites as 0.05:0.1 (~ 0.5), 0.05:0.1 (~ 0.5), and 0.025:0.1 (~ 0.25), respectively (denoted as ACA-500-S@PANi-45%, ACA-500-S@PANi-55%, and ACA-500-S@PANi-61%, respectively).

### Sample Characterization

The morphology and microstructure of samples were observed by using a field emission scanning electron microscopy (FESEM, JSM-6330F) and a transmission electron microscopy (TEM, Tecnai G2 Spirit). FTIR spectra were recorded using an Equinox 555 (Bruker, Germany) from 400 to 4000 cm^−1^ under 2 cm^−1^ resolution. Porosity was determined using a Micromeritics ASAP 2020 instrument at 77 K. The surface area measurements were analyzed according to the multipoint Brunauer-Emmett-Teller (BET) theory. The pore size distribution was calculated based on original density function theory (DFT). TGA (Netzsch TG-209) was carried out to determine the sulfur content in the composites. X-ray diffraction (XRD) patterns were recorded on a D-MAX 2200 VPC diffractometer using Cu Kα radiation (40 kV, 26 mA). Raman spectra were measured and collected using a laser micro-Raman spectrometer (Renishaw inVia) with 633-nm laser excitation under ambient conditions. X-ray photoelectron spectroscopy (XPS) measurements were performed on an ESCALab250 instrument.

### Electrochemical Measurements

Cathode slurry was fabricated by mixing 80 wt% ACA-500-S@PANi or ACA-500-S composite, 10 wt% Super P, and 10 wt% polyvinylidene fluoride in *N*-methyl pyrrolidone (NMP) solvent. Then, the slurry was spread onto a piece of carbon coating Al foil substrate. The obtained electrode film was dried at 60 °C for 12 h and punched into disks with a diameter of 12 mm. The Li-S cells were assembled with CR2032 coin-type cells with a carbon/sulfur cathode, commercial polypropylene separator (Celgard 2400), and lithium foil anode in an argon-filled glove box with moisture and oxygen content below 1.0 ppm. The electrolyte used was a freshly prepared solution of lithium bis(trifluoromethanesulfonyl)imide (1 M) in 1,2-dimethoxyethane (DME) and 1,3-dioxolane (DOL) (volume ratio = 1:1), including a LiNO_3_ (1 wt%) additive. Cyclic voltammogram (CV) was measured on a CHI660C electrochemical workstation at a scanning rate of 0.2 mV s^−1^ between 1.7 and 2.8 V. Galvanostatic charging/discharging tests were operated in the potential range of 1.7~2.8 V at room temperature by using a LAND CT2001A battery test system. Notably, all specific capacity data in this work were calculated only based on the mass of sulfur. Electrochemical impedance spectra (EIS) data of the cells were recorded by using a Zahner IM6ex electrochemical workstation, which was carried out in the frequency range of 100 kHz to 10 mHz at perturbation amplitudes of 5 mV.

## Results and Discussion

**Fig. 1:**
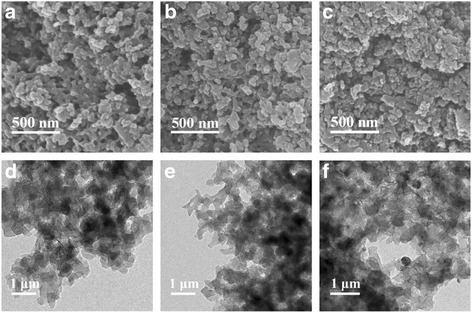
SEM images of **a** ACA-500, **b** ACA-500-S, and **c** ACA-500-S@PANi; TEM images of **d** ACA-500, **e** ACA-500-S, and **f** ACA-500-S@PANi

FESEM and TEM are used to investigate the morphologies of ACA-500, ACA-500-S, and ACA-500-S@PANi, and the images are shown in Fig. 1. It can be seen from Fig. 1a that the ACA-500 presents a three-dimensional crosslinked carbon network microstructure with nanoparticles of about 10~30 nm. After encapsulating sulfur and coating PANi, ACA-500-S and ACA-500-S@PANi exhibit similar morphologies (Fig. 1b, c) to ACA-500. No obvious large agglomerations are discovered on the surface of the ACA-500-S, indicating that the sulfur have diffused into the nanopores of ACA-500 during the heat-melting process. The morphology of ACA-500-S@PANi is almost the same as that of ACA-500-S, implying that the PANi is uniformly distributed on the surface of the ACA-500-S substrate. These results can also be verified by the TEM images (Fig. 1d–f). High-resolution TEM images of ACA-500-S and ACA-500-S@PANi composites combining the corresponding elemental mapping of carbon, sulfur, nitrogen, and oxygen (Additional file 1: Figures S1 and S2) also reveal a homogeneous distribution of sulfur among these composites. They also confirm that the PANi-coating layer does not change the sulfur distribution in the carbon substrate.

**Fig. 2:**
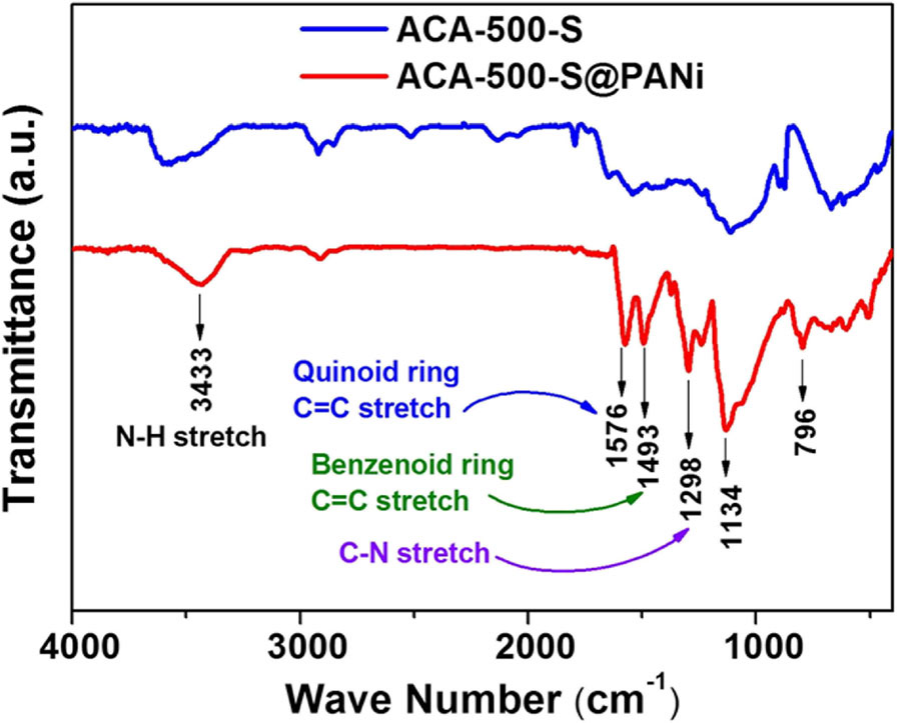
FTIR spectra of ACA-500-S and ACA-500-S@PANi composites

FTIR spectra of ACA-500-S and ACA-500-S@PANi composite are shown in Fig. 2, which fully match those results reported in the literatures [32]. In the ACA-500-S@PANi spectrum, the characteristic peaks at 1576 and 1493 cm^−1^ are attributed to vibrations of the quinoid ring and benzene ring, respectively. The wide peak at 3433 cm^−1^ is assigned to the secondary amines N–H stretching mode from the PANi. The other bands at 1298, 1134, and 796 cm^−1^ can be associated with the C–N stretching of the secondary aromatic amine, aromatic C–H in-plane bending, and out-of-plane bending vibration, respectively. These results confirm the successful coating of polyaniline on the surface of the ACA-500-S composite. Here, PANi as a soft buffer agent can bridge carbon and sulfur, enhance their intimate contact, and shorten charge transport distance and is believed to be capable of trapping polysulfides’ negative ions and accommodate volume expansion. Therefore, an improved electrochemical performance of Li-S batteries with ACA-500-S@PANi composite can be expected.

**Fig. 3:**
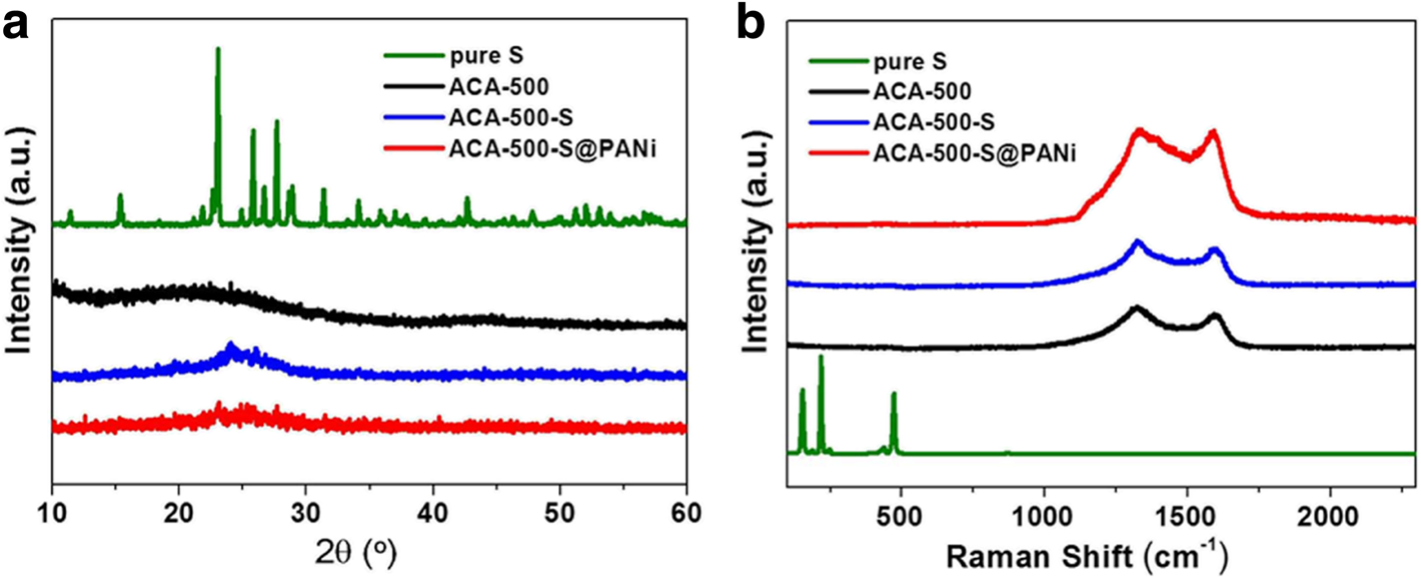
**a** XRD patterns and **b** Raman spectra of pure sulfur, ACA-500, ACA-500-S, and ACA-500-S@PANi composites

The ACA-500-S and ACA-500-S@PANi composites are further characterized by XRD and Raman (Fig. 3). Figure 3a shows the XRD patterns of pure sulfur, ACA-500, ACA-500-S, and ACA-500-S@PANi composites, respectively. Obviously, no crystalline peak associated with orthorhombic sulfur is observed either in the ACA-500-S or in the ACA-500-S@PANi composite, which reveals that the sulfur incorporates the pores of carbon matrix and exists as an amorphous state. A broad diffraction peak centered at 24° appeared in the XRD patterns of the ACA-500-S and ACA-500-S@PANi composites and is attributed to amorphous activated carbon aerogels. No visible signal below 500 cm^−1^ is found in Raman spectra (Fig. 3b), further demonstrating that sulfur is evenly dispersed. For the graphitization degree of carbon materials, the ratios of D-band and G-band intensities are calculated. The *I*_D_/*I*_G_ of ACA-500-S (1.17) is higher than that of ACA-500 (1.10), suggesting that disorder degree of the ACA-500 increases after the infiltration of sulfur [33].

XPS is performed to analyze the chemical status of sulfur and nitrogen in ACA-500-S and ACA-500-S@PANi composites (Additional file 1: Figures S3 and S4). The survey XPS spectrum of the ACA-500-S composite in Additional file 1: Figure S3a shows four peaks centered at 164.0, 228.9, 284.8, and 532.6 eV which are assigned to S 2p, S 1s, C 1s, and O 1s, respectively, indicating the presence of S, C, and O elements. The C 1s region spectrum in Additional file 1: Figure S3b displays one main peak at 284.8 eV, indicating the existence of C–C bonds and the amorphous characteristic of the ACA-500 substrate. The S 2p spectrum has been fitted and deconvoluted into two asymmetrical peaks at 164.0 and 165.2 eV, which correspond to the S 2p_3/2_ and S 2p_1/2_, respectively, identified with standard energy separation of 1.2 eV between the S 2p_3/2_ and S 2p_1/2_ (163.6 and 164.8 eV) spin orbit levels [34–36]. The N 1s region spectrum of ACA-500-S@PANi in Additional file 1: Figure S4b is deconvoluted into two component peaks at 399.8 eV (–NH–) and 401.1 eV (–NH^+^), suggesting the emeraldine salt state of the coated PANi onto the surface of ACA-500-S@PANi [37].

**Fig. 4:**
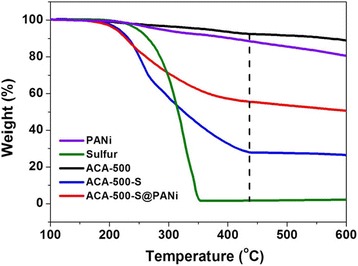
TGA curves of PANi, pure sulfur, ACA-500, ACA-500-S, and ACA-500-S@PANi composites

**Fig. 5:**
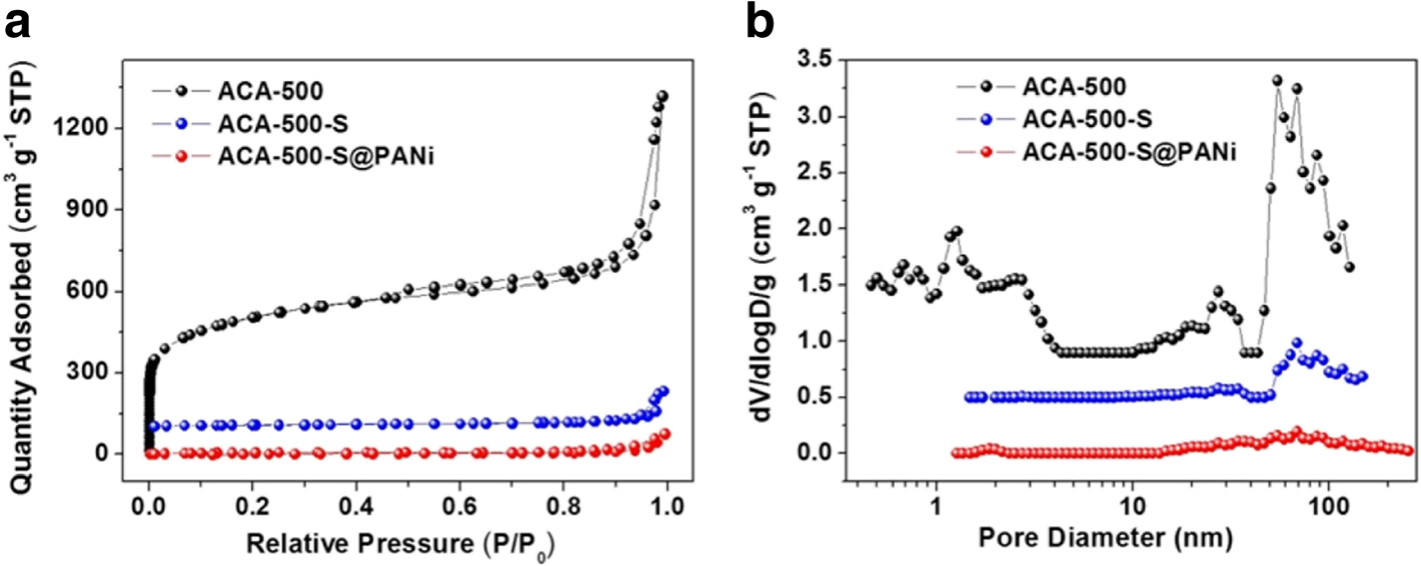
**a** N_2_ adsorption-desorption isotherms and **b** pore size distribution curves of ACA-500, ACA-500-S, and ACA-500-S@PANi

The sulfur contents of ACA-500-S and ACA-500-S@PANi composites are determined from the thermogravimetric analysis. Figure 4 showed the TGA curves of pure sulfur, PANi, ACA-500, ACA-500-S, and ACA-500-S@PANi in N_2_ flow, indicating pure sulfur burns completely at around 350 °C and this temperature is delayed to 430 °C in ACA-500-S and ACA-500-S@PANi composites, implying the confinement effect of nanoporous carbon materials to sulfur. The sulfur content in the ACA-500-S and ACA-500-S@PANi composites were calculated to approximately 63 and 37.4%, respectively. The nitrogen adsorption-desorption isotherms are employed to examine the pore characteristics of ACA-500, ACA-500-S, and ACA-500-S@PANi composites (Fig. 5, Additional file 1: Table S1). As shown in Fig. 5a, ACA-500 exhibits type IV isotherm in the IUPAC classification with a typical mesopore hysteresis loop [38]. A very high nitrogen adsorption at low relative pressure reveals the presence of tremendous micropores within the carbon frameworks. The adsorption curves rise gradually and do not reach a plateau near the *P*/*P*_0_ of 1.0, implying the existence of numerous interval mesopores. This conclusion is also verified by the pore size distribution curves based on DFT theory. The pore size distribution curve of ACA-500 shows that micropores locate about 1.3 nm and mesopores center at 2.6 and 27 nm (Fig. 5b). As shown in Additional file 1: Table S1, the ACA-500 presents a high surface area of 1765 m^2^ g^−1^ and a large pore volume of 2.04 cm^3^ g^−1^. The specific surface area and pore volume of the ACA-500-S and ACA-500-S@PANi composites are obviously decreased to 31 m^2^ g^−1^ and 0.207 cm^3^ g^−1^ and 26 m^2^ g^−1^ and 0.116 cm^3^ g^−1^ after infiltrating sulfur and coating PANi. The pore size distribution curves indicate that the pores in ACA-500-S and ACA-500-S@PANi decrease or disappear, indicating that sulfur have incorporated into the nanopores of ACA-500 and the PANi and have uniformly coated on the surface of ACA-500-S.

**Fig. 6:**
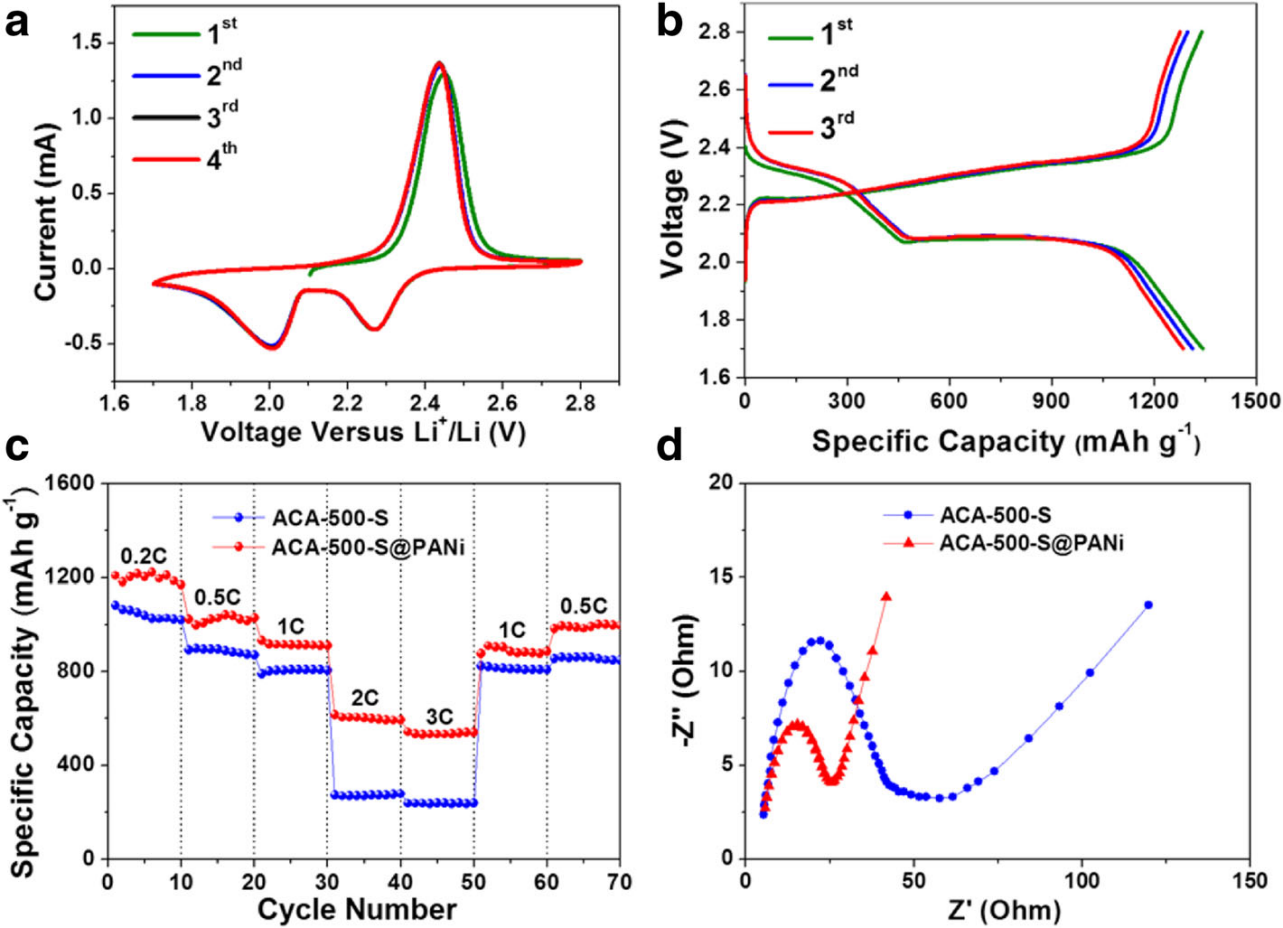
**a** The first four cyclic voltammogram curves at 0.2 mV s^−1^ and **b** the first three charge/discharge curves at 0.1 C of ACA-500-S@PANi cathodes; **c** rate performances and **d** EIS spectra of ACA-500-S and ACA-500-S@PANi cathodes

**Fig. 7:**
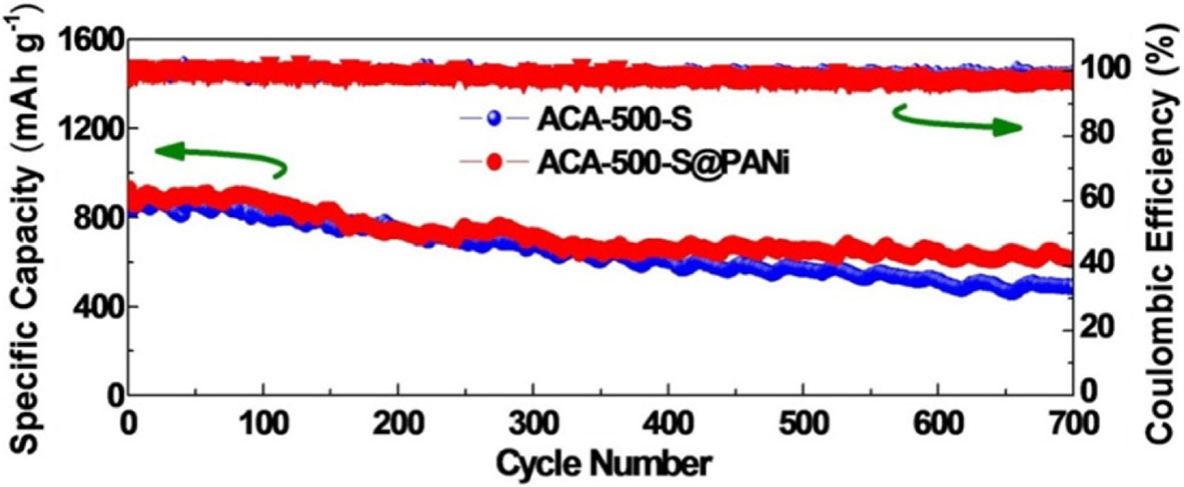
Cycle performance of ACA-500-S and ACA-500-S@PANi cathodes at 1C

The electrochemical performances of ACA-500-S@PANi composites are shown in Figs. 6 and 7, and that of ACA-500-S composites is simultaneously given for comparison. In the first four cyclic voltammogram curves of ACA-500-S@PANi composites at 0.2 mV s^−1^ (Fig. 6a), two main reduction peaks at ~ 2.27 and ~ 2.01 V (vs. Li/Li^+^) are observed during the cathodic scan, which correspond to a two-step reduction reaction from sulfur to long-chain polysulfides (Li_2_S_*x*_, 4 < *x* ≤ 8) and further to Li_2_S_2_/Li_2_S. The oxidation peak at ~ 2.34 V is attributed to oxidation of Li_2_S_2_/Li_2_S to long polysulfides or sulfur. Figure 6b shows the first three charge/discharge profiles for ACA-500-S@PANi composites at 0.1C between 1.7 and 2.8 V. The discharge curves present two typical plateaus, which could be assigned to the two-step reaction of element sulfur during the discharge process, which is consistent with the CV measurement results. The almost overlapping upper discharge plateaus demonstrated rare active material loss and high electrochemical stability in this process. The lower discharge plateau decreased steeply, which indicated slow reaction kinetic, partial polysulfide dissolution, and active material loss for ACA-500-S@PANi composites in this stage. The rate performances of ACA-500-S and ACA-500-S@PANi composites are evaluated by applying progressive current rates from 0.2 to 3C for 10 cycles at each current density, as shown in Fig. 6c and Additional file 1: Figure S5. Obviously, the ACA-500-S@PANi electrode delivers high specific capacities of 1208, 1022, 933, 616, and 542 mAh g^−1^ at 0.2, 0.5, 1, 2, and 3C, respectively. These capacity values are superior to that of ACA-500-S electrode which only delivers discharge capacities of 1082, 893, 790, 272, and 237 mAh g^−1^. When the current density is returned from 3 to 1C and 0.5C, the original capacities are almost completely recovered. Discharge capacities of 877 mAh g^−1^ at 1C and 982 mAh g^−1^ at 0.5C are obtained for ACA-500-S@PANi electrode after 50 cycles, indicating excellent rate performances and high stability of electrode. Figure 6d shows the electrochemical impedance spectra of the freshly prepared ACA-500-S and ACA-500-S@PANi electrodes. Nyquist plots of these two electrodes are composed of a semicircle in the high- and medium-frequency region corresponding to charge transfer resistance and an inclined line in the low-frequency region reflecting the ion diffusion resistance within the electrode. Distinctly, the ACA-500-S@PANi composite exhibits a lower charge transfer resistance than that of ACA-500-S, which can be attributed to introduction of the highly conductive polyaniline-coating layer, offering an effective electron transport path.

To further investigate improved electrochemical reversibility and cycling performance, a prolonged cycling test for the ACA-500-S and ACA-500-S@PANi composites is carried out at 1C (Fig. 7, Additional file 1: Figure S6). The ACA-500-S@PANi composite delivers a high initial discharge capacity of 926 mAh g^−1^ and still maintains a reversible capacity of 615 mAh g^−1^ after 700 cycles with capacity retention ratio of 66.4% and decay rate of 0.48‰ per cycle. By contrast, ACA-500-S exhibits an initial discharge capacity up to 916 mAh g^−1^ and still retains 493 mAh g^−1^ over 700 cycles with capacity retention of 53.8% and decay of 0.66‰ per cycle. Coulombic efficiency of the two composites during the whole cycling maintains near 99%. Moreover, to prove that improvement of lithium-sulfur battery performance for ACA-500-S@PANi compared with ACA-500-S is attributed to the PANi coating, rather than the low sulfur content, we further examine the electrochemical performance for a couple of ACA-500-S@PANi-55% and ACA-500-S-54% composites with similar sulfur contents (55 vs 54%, Additional file 1: Figure S7). As shown in Additional file 1: Figure S8a–c, ACA-500-S@PANi-55% presents superior rate performance than ACA-500-S-54% and delivers reversible capacities of 1109, 880, 741, 602, and 447 mAh g^−1^ at 0.2, 0.5, 1, 2, and 3C, respectively, greater than that of 921, 693, 580, 499, and 402 mAh g^−1^ for ACA-500-S-54%, confirming the advantage of PANi coating. Benefiting from the PANi coating, the ACA-500-S@PANi composites demonstrate superior stability when further increasing the sulfur contents. As shown in Additional file 1: Figure S8d, the ACA-500-S@PANi-45% delivers an initial capacity of 815 mAh g^−1^ and maintains a reversible capacity of 687 mAh g^−1^ at 1C rate after 100 cycles with a capacity retention ratio of 84.3%. The ACA-500-S@PANi-61% delivers 611 mAh g^−1^ of initial capacity and maintains 416 and 394 mAh g^−1^ of reversible capacities after 100 and 120 cycles with capacity retention ratios of 68.1 and 64.5%, respectively. Such excellent cycling stabilities for the ACA-500-S@PANi-45% and ACA-500-S@PANi-61% have exceeded many other PANi-coated carbon/sulfur composites with similar sulfur contents reported in previous works (Additional file 1: Table S2).

Overall, the enhanced electrochemical performance of ACA-500-S@PANi composites should be attributed to the synergistic effect on the excellent conductivity from both the activated carbon aerogel framework in the matrix and the PANi-coating layer on the surface. The ACA-500 framework with a high surface area and unique 3D interconnected hierarchical porous structure offers an efficient conductive network for sulfur and highly conductive PANi-coating layer, which not only acts as the link between carbon and sulfur, enhances their intimate contact, and shortens ion and electron conduction path but also prevents the dissolution of polysulfide species and accommodates volume change during the charge/discharge process.

## Conclusions

In summary, polyaniline-coated activated carbon aerogel/sulfur composites (ACA-500-S@PANi) are successfully prepared by in situ chemical oxidation polymerization of aniline on the surface of the ACA-500-S composite obtained from the thermal treatment of sulfur and ACA-500. ACA-500 with a high surface area (1765 m^2^ g^−1^) and hierarchical porous nanonetwork structure was synthesized through a KOH activation method from organic aerogel (RC-500). ACA-500-S@PANi composite exhibits more excellent electrochemical performance than ACA-500-S composite. It shows a high reversible capacity of 1208 mAh g^−1^ at 0.2C and retains 542 mAh g^−1^ even at a high rate (3C). Furthermore, it delivers an initial discharge capacity of 926 mAh g^−1^ and exhibits excellent capacity retention of 66.4% (615 mAh g^−1^) and extremely low capacity decay rate (0.48‰ per cycle) after 700 cycles at 1C. The remarkably improved electrochemical performance of the ACA-500-S@PANi composite should be attributed to its high surface area, unique 3D interconnected hierarchical porous carbon network framework, and highly conductive PANi-coating layer. The PANi layer not only acts as a link between carbon and sulfur and enhances their intimate contact, but also provides strong physical and chemical confinement to polysulfides and minimizes the active material loss, shortens ion and electron transport distance, and further improves the conductivity of the composite.

## Additional File

Additional file 1: Supporting information. **Figure S1.** TEM images of (a) ACA-500-S and the corresponding elemental mapping for (b) carbon, (c) sulfur, (d) oxygen. **Figure S2.** STEM images of (a) ACA-500-S@PANi and the corresponding elemental mapping for (b) carbon, (c) nitrogen, (d) sulfur, and (d) oxygen. **Figure S3.** The total XPS spectra of (a) ACA-500-S, (b) C 1s, and (c) S 2p spectra of ACA-500-S. The peaks at 164.0 and 165.2 eV in (c) indicate that the uniformly encapsulated sulfur exists in the form of elemental sulfur. **Figure S4.** (a) The total XPS spectra and (b) N 1s spectrum of ACA-500-S@PANi. **Figure S5.** Discharge-charge curves at various rates for (a) ACA-500-S@PANi and (b) ACA-500-S cathodes. **Figure S6.** Discharge-charge curves recorded at different cycles for (a) ACA-500-S@PANi and (b) ACA-500-S cathodes at 1C. **Figure S7.** TGA curves of (a) ACA-500-S-70% (black), ACA-500-S@PANi-61% (blue), and ACA-500-S@PANi-55% (red) and (b) ACA-500-S-54% (violet) and ACA-500-S@PANi-45% (olive). **Figure S8.** (a) Rate performances of ACA-500-S-54% and ACA-500-S@PANi-55% cathodes. Discharge-charge curves at various rates for (b) ACA-500-S@PANi-55% and (c) ACA-500-S-54% cathodes. (d) Cycle performances of ACA-500-S@PANi-45% and ACA-500-S@PANi-61% cathodes at 1C. **Table S1.** Textual characteristic of ACA-500, ACA-500-S, and ACA-500-S@PANi. **Table S2.** Summary of cycle stability performances of representative conductive PANi coating for carbon/S cathodes at 1 C rate. (DOCX 980 kb)

## Abbreviations

ACA-500: Activated carbon aerogels
ACA-500-S: Activated carbon aerogel/sulfur composite
ACA-500-S@PANi: Polyaniline-coated activated carbon aerogel/sulfur composite
CAs: Carbon aerogels
CV: Cyclic voltammogram
FESEM: Field emission scanning electron microscopy
FTIR: Fourier transform infrared spectroscopy
PANi: Polyaniline
RC-500: Organic aerogel
TEM: Transmission electron microscopy
TGA: Thermogravimetric analysis
XPS: X-ray photoelectron spectroscopy
XRD: X-ray diffraction
