# Supplementary material for: Polyaniline-Coated Activated Carbon Aerogel/Sulfur Composite for High-performance Lithium-Sulfur Battery
Source: Nanoscale Res Lett. 2017 Dec 12;12:617. doi: 10.1186/s11671-017-2372-6 (PMC5727006; doi:10.1186/s11671-017-2372-6)
Supplement: Additional file 1: — Supporting information. Figure S1. TEM images of (a) ACA-500-S and the corresponding elemental mapping for (b) carbon, (c) sulfur, (d) oxygen. Figure S2. STEM images of (a) ACA-500-S@PANi and the corresponding elemental mapping for (b) carbon, (c) nitrogen, (d) sulfur, and (d) oxygen. Figure S3. The total XPS spectra of (a) ACA-500-S, (b) C 1s, and (c) S 2p spectra of ACA-500-S. The peaks at 164.0 and 165.2 eV in (c) indicate that the uniformly encapsulated sulfur exists in the form of elemental sulfur. Figure S4. (a) The total XPS spectra and (b) N 1s spectrum of ACA-500-S@PANi. Figure S5. Discharge-charge curves at various rates for (a) ACA-500-S@PANi and (b) ACA-500-S cathodes. Figure S6. Discharge-charge curves recorded at different cycles for (a) ACA-500-S@PANi and (b) ACA-500-S cathodes at 1C. Figure S7. TGA curves of (a) ACA-500-S-70% (black), ACA-500-S@PANi-61% (blue), and ACA-500-S@PANi-55% (red) and (b) ACA-500-S-54% (violet) and ACA-500-S@PANi-45% (olive). Figure S8. (a) Rate performances of ACA-500-S-54% and ACA-500-S@PANi-55% cathodes. Discharge-charge curves at various rates for (b) ACA-500-S@PANi-55% and (c) ACA-500-S-54% cathodes. (d) Cycle performances of ACA-500-S@PANi-45% and ACA-500-S@PANi-61% cathodes at 1C. Table S1. Textual characteristic of ACA-500, ACA-500-S, and ACA-500-S@PANi. Table S2. Summary of cycle stability performances of representative conductive PANi coating for carbon/S cathodes at 1 C rate. (DOCX 980 kb) [file 11671_2017_2372_MOESM1_ESM.docx]

**[Supporting information](http://www.nature.com/nmat/journal/v8/n7/suppinfo/nmat2469_S1.html)**

**Polyaniline-coated activated carbon aerogels/sulfur composite for high performance lithium-sulfur battery**

Zhiwei Tang, Jinglin Jiang, Shaohong Liu, Luyi Chen, Ruliang Liu, Bingna Zheng, Ruowen Fu^*^, Dingcai Wu^*^

Materials Science Institute, PCFM Lab and GDHPRC Lab, School of Chemistry, Sun Yat-sen University, Guangzhou 510275, P. R. China

Corresponding authors: Tel./Fax: +86-020-84110695

E-mail: cesfrw@mail.sysu.edu.cn; [wudc@mail.sysu.edu.cn](mailto:wudc@mail.sysu.edu.cn)


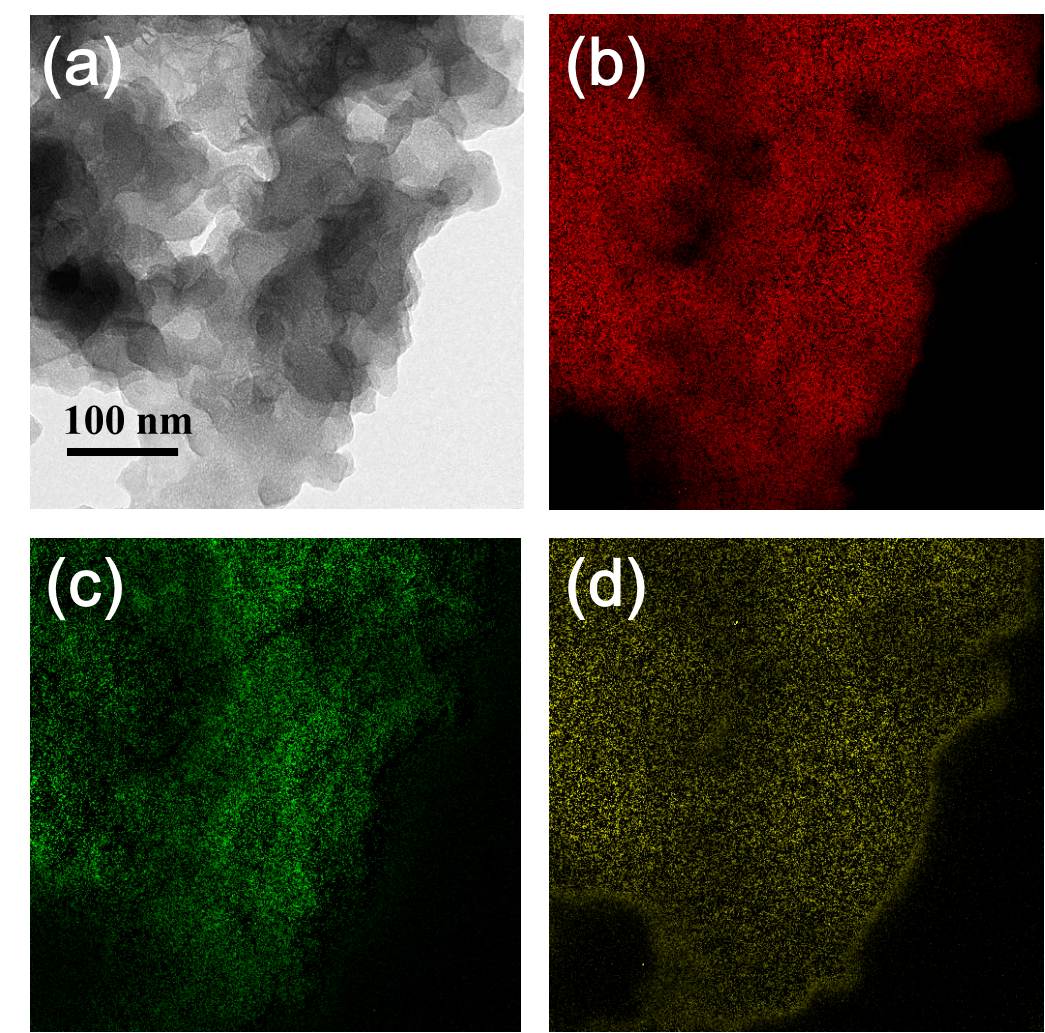


**Figure S1.** TEM images of (a) ACA-500-S and the corresponding elemental mapping for (b) carbon, (c) sulfur, (d) oxygen.


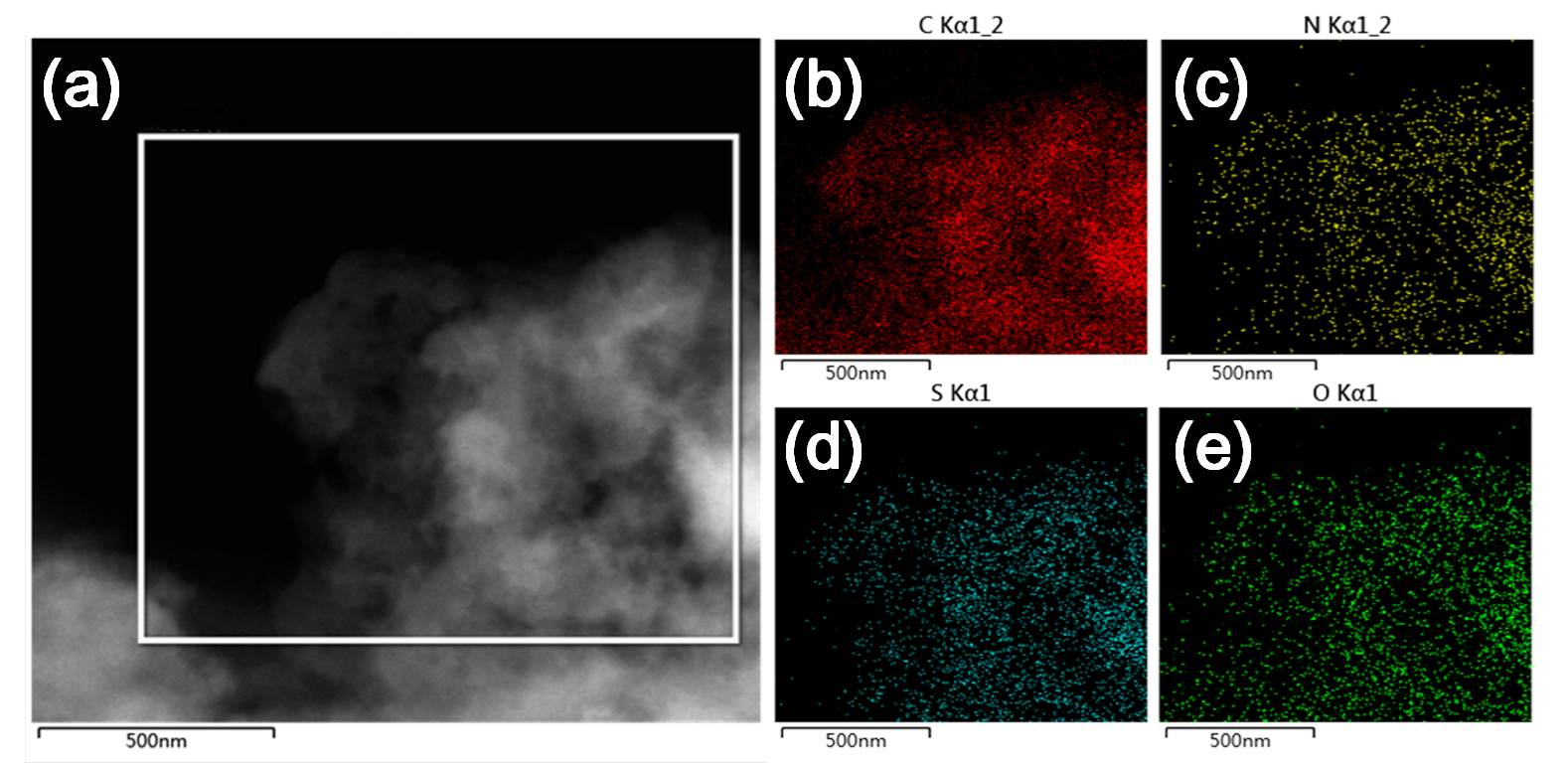


**Figure S2.** STEM images of (a) ACA-500-S@PANi and the corresponding elemental (c) nitrogen, (d) sulfur and (d) oxygen.


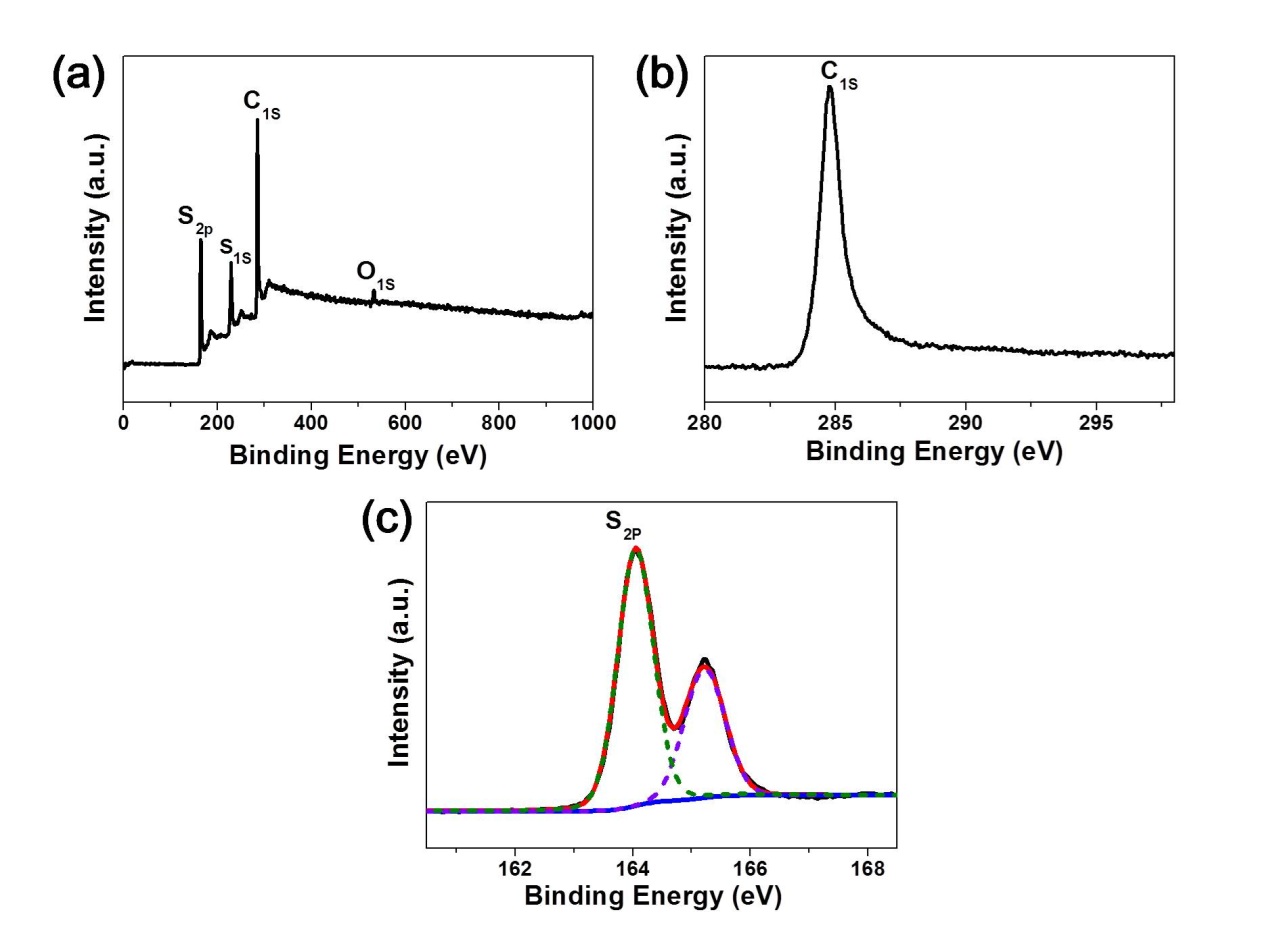


**Figure S3.** The total XPS spectra of (a) ACA-500-S; (b) C 1s and (c) S 2p spectra of ACA-500-S. The peaks at 164.0 and 165.2 eV in (c) indicate that the uniformly encapsulated sulfur exists in the form of elemental sulfur.


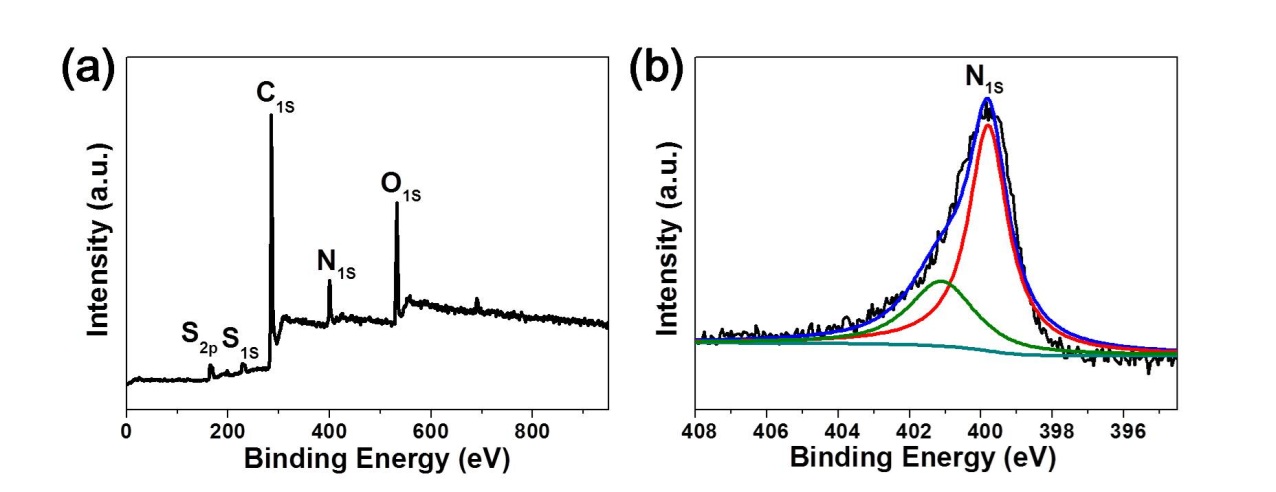


**Figure S4.** (a) The total XPS spectra and (b) N 1s spectrum of ACA-500-S@PANi.


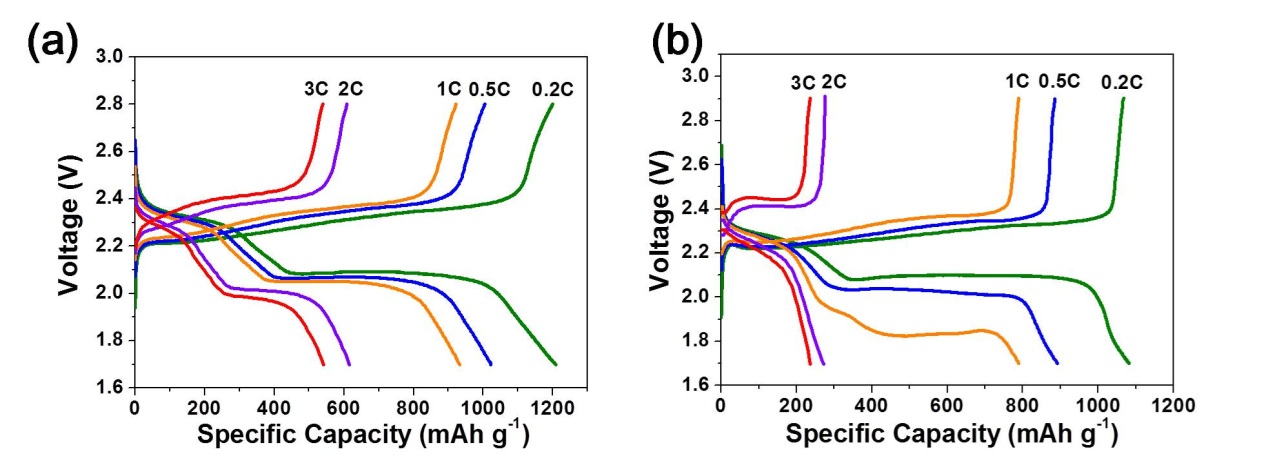


**Figure S5.** Discharge-charge curves at various rates for (a) ACA-500-S@PANi and (b) ACA-500-S cathodes.


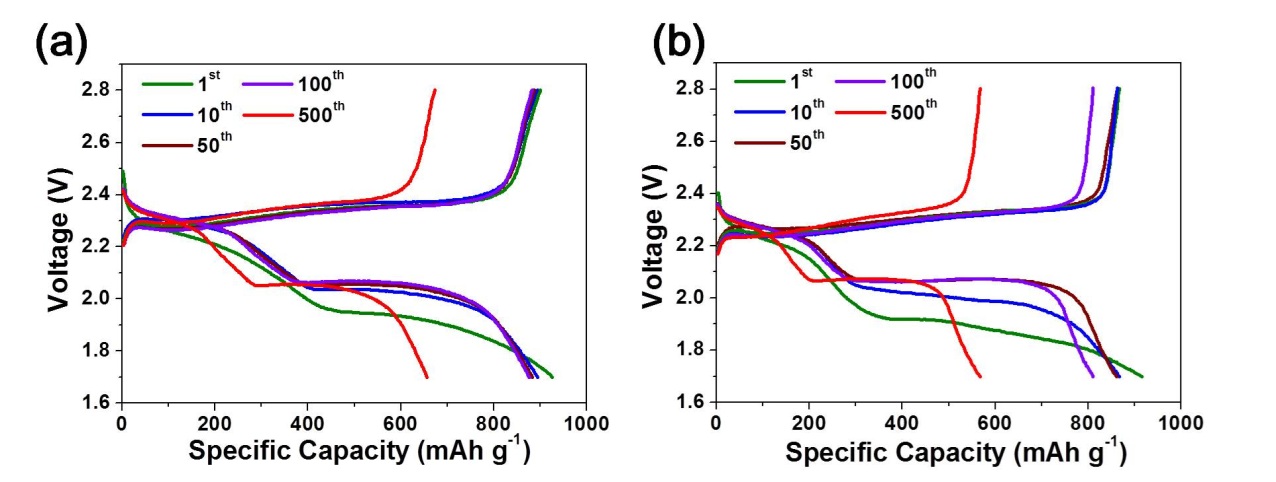


**Figure S6.** Discharge-charge curves recorded at different cycles for (a) ACA-500-S@PANi and (b) ACA-500-S cathodes at 1C.


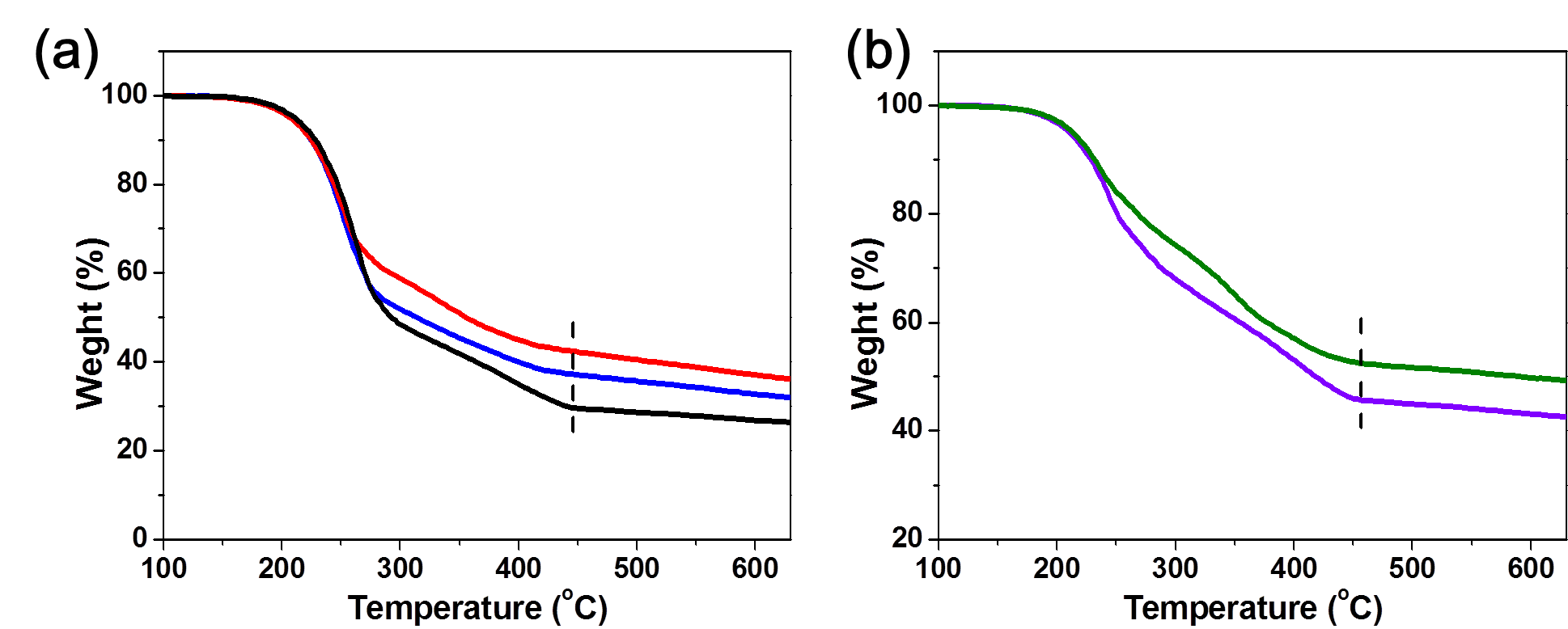


**Figure S7.** TGA curves of (a) ACA-500-S-70% (black), ACA-500-S@PANi-61% (blue) and ACA-500-S@PANi-55% (red), (b) ACA-500-S-54% (violet) and ACA-500-S@PANi-45% (olive).


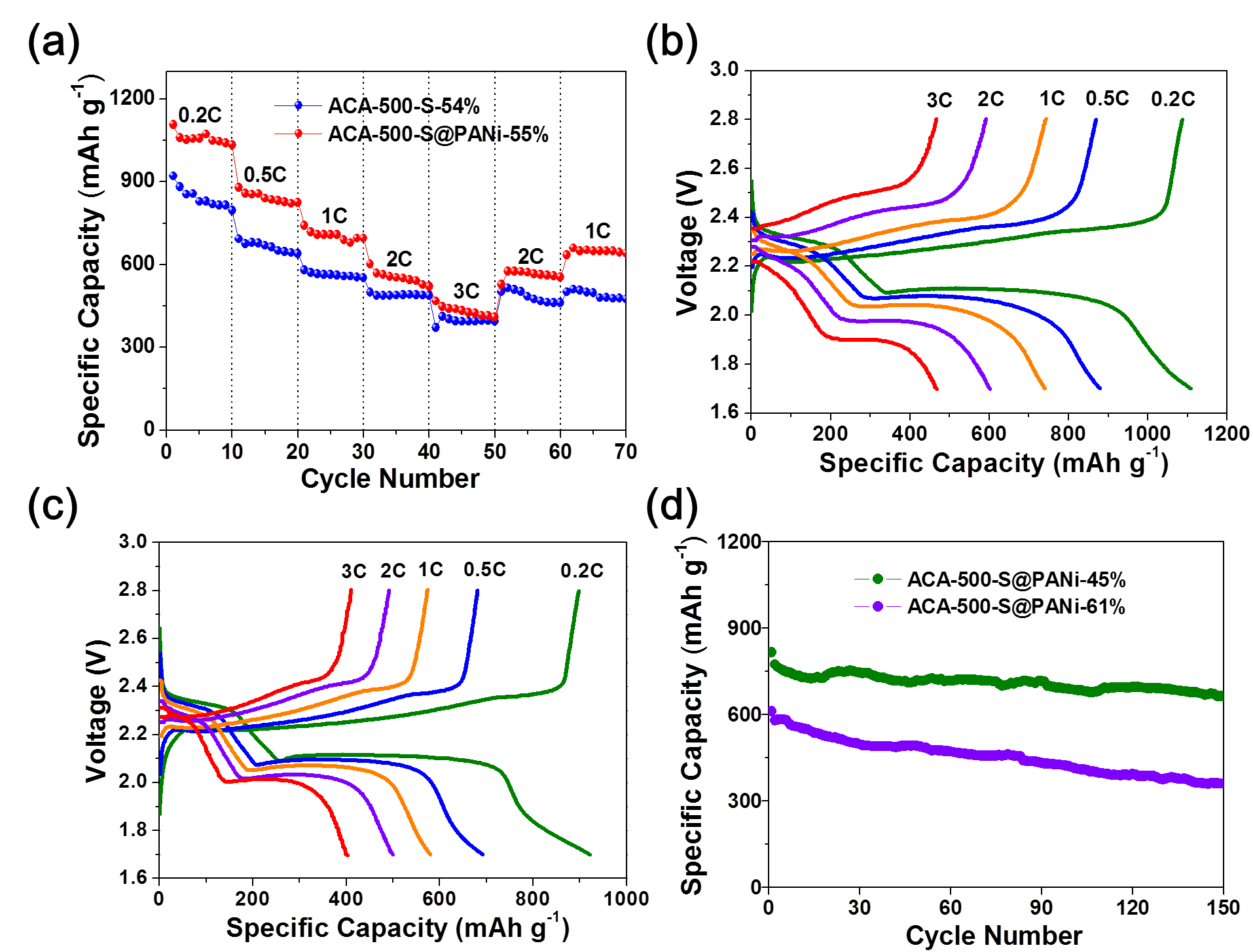


**Figure S8.** (a) Rate performances of ACA-500-S-54% and ACA-500-S@PANi-55% cathodes. Discharge-charge curves at various rates for (b) ACA-500-S@PANi-55% and (c) ACA-500-S-54% cathodes. (d) Cycle performances of ACA-500-S@PANi-45% and ACA-500-S@PANi-61% cathodes at 1C.

**Table. S1.** Textual characteristic of ACA-500, ACA-500-S and ACA-500-S@PANi

| Sample | S_BET_/m^2^ g^-1^ | S_mic_/m^2^ g^-1^ | V_total_/cm^3^ g^-1^ | V_mic_/cm^3^ g^-1^ | V_ext_/cm^3^ g^-1^ |
| --- | --- | --- | --- | --- | --- |
| ACA-500 | 1765 | 697 | 2.04 | 0.310 | 1.68 |
| ACA-500-S | 31 | / | 0.207 | / | 0.217 |
| ACA-500-S@PANi | 26 | / | 0.116 | / | 0.109 |

**Table. S2.** Summary of cycle stability performances of representative conductive PANi coating for carbon/S cathodes at 1 C rate.

| **Sample** | **Sulfur content** | **Cycle number** | **Capacity retention ratio (%)** | **Ref.** |
| --- | --- | --- | --- | --- |
| ACA-500-S@PANi | 45% | 100 | **84.3** | This work |
|  | 61% | 100 | **68.1** |  |
|  |  | 120 | **64.5** |  |
| CMK3/S-PANi | 41% | 100 | **58.8** | 1 |
| PANi@S/C | 43.7% |  | **42.4** | 2 |
| PUVGCF/S/PANi | 60% |  | **56.2** | 3 |
| SPKB-30/S  (S/PANi-KB) | 57% | 120 | **56.4** | 4 |

**Supplementary References**

1. Jin J, Wen Z, Ma G, Lu Y, Rui K (2014) Mesoporous carbon/sulfur composite with polyaniline coating for lithium sulfur batteries. Solid State Ionics 262:170-173

2. Li G C, Li G R, Ye S H, Gao X P (2012) A Polyaniline-Coated Sulfur/Carbon Composite with an Enhanced High-Rate Capability as a Cathode Material for Lithium/Sulfur Batteries. Adv Energy Mater 2(10):1238-1245

3. Zhou L, Mao H, Yu A (2016) Polyaniline-coated partially unzipped vapor-grown carbon fibers/sulfur microsphere composites for Li-S cathodes. J Electroanal Chem 761:62-67

4. Zhao X, Ahn H J, Kim K W, Cho K K, Ahn J H (2015) Polyaniline-Coated Mesoporous Carbon/Sulfur Composites for Advanced Lithium Sulfur Batteries. J Phys Chem C 119(15):7996-8003
